# TBX2 represses CST6 resulting in uncontrolled legumain activity to sustain breast cancer proliferation: a novel cancer-selective target pathway with therapeutic opportunities.

**DOI:** 10.18632/oncotarget.1707

**Published:** 2014-02-08

**Authors:** Zenobia C. D'Costa, Catherine Higgins, Chee Wee Ong, Gareth W. Irwin, David Boyle, Darragh G. McArt, Karen McCloskey, Niamh E. Buckley, Nyree T. Crawford, Lalitha Thiagarajan, James T. Murray, Richard D. Kennedy, Karl A. Mulligan, D. Paul Harkin, David J.J. Waugh, Chris J. Scott, Manuel Salto-Tellez, Richard Williams, Paul B. Mullan

**Affiliations:** ^1^ Centre for Cancer Research and Cell Biology, Queen's University Belfast, Belfast, UK; ^2^ Biomedical Science Institute, Trinity College Dublin, College Green, Dublin 2, Ireland; ^3^ School of Biological Sciences, Queen's University Belfast, Belfast, UK; ^4^ Northern Ireland Science Park, Belfast, UK; ^5^ School of Pharmacy, Queen's University Belfast, Belfast, UK

**Keywords:** TBX2, CST6, LGMN, breast cancer

## Abstract

TBX2 is an oncogenic transcription factor known to drive breast cancer proliferation. We have identified the cysteine protease inhibitor Cystatin 6 (CST6) as a consistently repressed TBX2 target gene, co-repressed through a mechanism involving Early Growth Response 1 (EGR1). Exogenous expression of CST6 in TBX2-expressing breast cancer cells resulted in significant apoptosis whilst non-tumorigenic breast cells remained unaffected. CST6 is an important tumor suppressor in multiple tissues, acting as a dual protease inhibitor of both papain-like cathepsins and asparaginyl endopeptidases (AEPs) such as Legumain (LGMN). Mutation of the CST6 LGMN-inhibitory domain completely abrogated its ability to induce apoptosis in TBX2-expressing breast cancer cells, whilst mutation of the cathepsin-inhibitory domain or treatment with a pan-cathepsin inhibitor had no effect, suggesting that LGMN is the key oncogenic driver enzyme. LGMN activity assays confirmed the observed growth inhibitory effects were consistent with CST6 inhibition of LGMN. Knockdown of LGMN and the only other known AEP enzyme (GPI8) by siRNA confirmed that LGMN was the enzyme responsible for maintaining breast cancer proliferation. CST6 did not require secretion or glycosylation to elicit its cell killing effects, suggesting an intracellular mode of action. Finally, we show that TBX2 and CST6 displayed reciprocal expression in a cohort of primary breast cancers with increased TBX2 expression associating with increased metastases. We have also noted that tumors with altered TBX2/CST6 expression show poor overall survival. This novel TBX2-CST6-LGMN signaling pathway, therefore, represents an exciting opportunity for the development of novel therapies to target TBX2 driven breast cancers.

## INTRODUCTION

TBX2 is a member of the T-box family of transcription factors, which play important roles in developmental gene regulation [1]. T-box proteins bind DNA and regulate gene expression through highly conserved 180-200 amino acid T-box motifs. TBX2 is an embryonically restricted gene normally expressed in the milk ridge during the development of the duct system of the mammary gland in the mouse [1]. TBX2 was first linked to cancer through its ability to facilitate senescence bypass in *Bmi^-/-^* mouse embryo fibroblasts [2] and was found to be a potent immortalizing gene downregulating *Cdkn2a* (*p19^ARF^*, *p14*^ARF^ in humans). TBX2 was also shown to bind to and repress the p21^WAF1^ promoter both *in vitro* and in *vivo* [3] and to have a role in maintaining proliferation and suppressing cell senescence in melanoma cells [4]. It promotes anchorage-independent growth and bypass of apoptotic pathways in adrenocortical carcinomas [5]. The Retinoblastoma protein Rb1 is another key tumor suppressor gene whose function is altered by TBX2 resulting in cell cycle perturbations [6]. We have previously shown addiction of TBX2-amplified cell lines to elevated TBX2 protein expression and that TBX2 transcriptionally represses the breast tumor suppressor gene N-myc Down Regulated Gene 1 (NDRG1) through interaction with EGR1 to promote cell proliferation and inhibit cellular senescence [7]. Oncogene addiction is thought to arise from dependence on a specific oncogenic pathway due to deactivation of an analogous redundant pro-survival pathway [8]. The oncogene is therefore not required for normal growth prior to its aberrant activation. This is usually evident from the lack of addiction to the oncogene in cell lines derived from corresponding normal tissue [reviewed in [8]]. In addition, some oncogenic events can generate a requirement of secondary oncogenic addictions [8, 9].

Cystatin 6 (CST6), also called cystatin E/M, was first identified as a cysteine protease inhibitor which showed a significant downregulation in breast cancer mRNA samples compared to matched normal control mRNAs [10]. Cystatins constitute a large family of protease inhibitors known to target lysosomal cysteine proteases and Asparaginyl Endopeptidases (AEPs). CST6 has been postulated to be a tumor suppressor gene in breast tissue [11], reducing breast cancer cell proliferation, migration, matrigel invasion, and adhesion to endothelial cells [12]. More recently, loss of CST6 expression has been reported in a number of other cancer types including cervical, glioma, prostate and gastric cancers [13],[14],[15],[16]. Loss of CST6 expression in breast cancers has been attributed to promoter hypermethylation [11]. CST6 has been shown to be important for skin differentiation which was shown to be disrupted in *cst6*-deficient mice, accompanied by uncontrolled activity of a specific AEP called Legumain (LGMN) [17]. LGMN is a known oncogene, an indicator of poor prognosis in colorectal and breast cancers and has been reported to be overexpressed in the majority of human solid tumors [18], [19].

In this study we identify CST6 as a novel TBX2 repressed target through a mechanism involving EGR1. We show that exogenous expression of wild-type CST6 induces apoptotic effects in TBX2 expressing breast cancer cells but not in non-tumorigenic breast cells. CST6-induced apoptosis is dependent on inhibition of the protease LGMN since CST6 mutants lacking LGMN (but not cathepsin) inhibitory activity were also unable to induce apoptosis. We also demonstrate that the secretion or glycosylation of CST6 was not required for this cell killing effect. We conclude that CST6 represents an important barrier to breast tumorigenesis which is targeted and bypassed by TBX2 transcriptional repression and we discuss the implications of these findings for breast tumorigenesis and the potential for novel therapeutic opportunities.

## RESULTS

**Figure 1:**
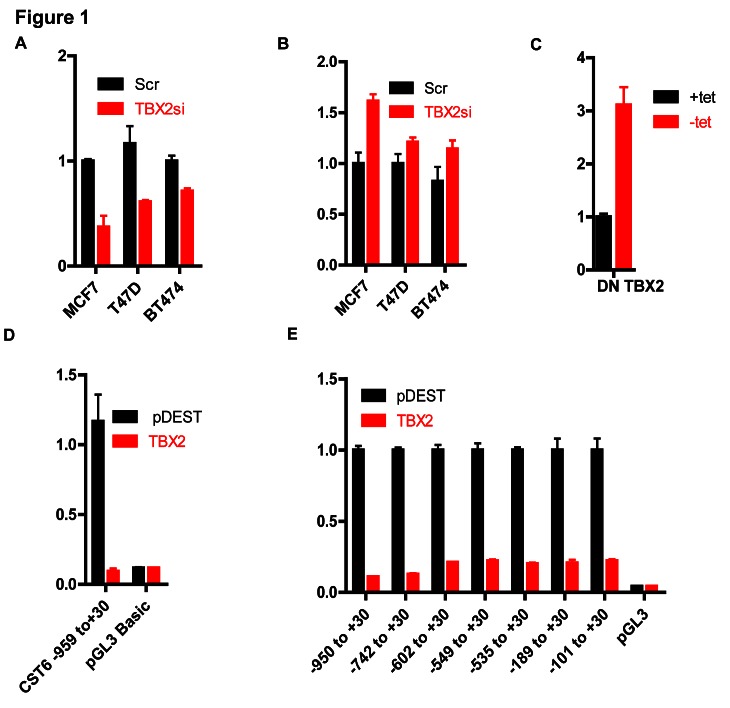
TBX2 represses the CST6 proximal promoter (A) RqPCR analysis showing TBX2 mRNA levels in MCF7, T47D, and BT474 cells transiently transfected with TBX2 siRNA (TBX2si) or scrambled control (Scr). GAPDH mRNA was used for normalization. (B) RqPCR analysis showing CST6 expression of the same samples in (A). (C) RqPCR analysis showing CST6 expression in MCF7 cells stably transfected with a tetracycline responsive FLAG tagged Dominant Negative TBX2 (Flag DN-TBX2) with GAPDH mRNA used for normalization. (D) Luciferase reporter assay of U2OS cells transiently transfected with the indicated CST6 promoter constructs or empty vector (pGL3 basic), either in presence of an empty vector (pDEST) or a TBX2 expression construct (TBX2). (E) Luciferase reporter assay of U2OS cells transiently transfected with a series of CST6 promoter constructs generated by exonuclease mapping or empty vector (pGL3 basic), either in the presence of pDEST or TBX2 expression constructs (TBX2).

CST6 was identified as a strongly repressed TBX2 transcriptional target in a microarray analysis of MCF7 cells following TBX2 siRNA knockdown (data not shown). Figures 1A and 1B show RqPCR values for TBX2 and CST6, respectively, following siRNA knockdown of TBX2 in three different breast cancer cell lines. CST6 was also upregulated in MCF7 cells following tetracycline-induced (-tet) expression of a truncated dominant negative TBX2 (DN-TBX2, Figure 1C, Supplementary Figure S1.A), missing the putative C-terminal repression domain [7]. Conversely, CST6 was downregulated following exogenous expression of wild-type TBX2 in U2OS cells (lacking endogenous TBX2 expression, Supplementary Figure S1.B and S1.C). To help define mechanistically how TBX2 repressed CST6 we generated a luciferase reporter construct of the CST6 proximal promoter (-959/+30). This construct was TBX2-responsive (Figure 1D), showing strong repression of luciferase following exogenous expression of TBX2 in U2OS cells. Exonuclease mapping of the -959/+30 reporter showed that even the shortest region (-101/+30 upstream of the CST6 transcriptional start site) was TBX2 responsive (Figure 1E).

**Figure 2:**
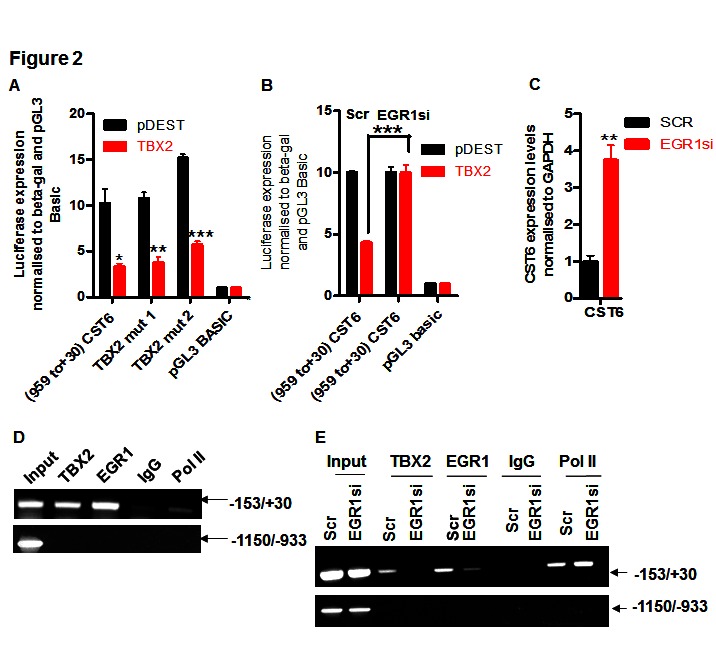
TBX2 represses CST6 through an EGR1-dependent mechanism (A) Luciferase reporter assay of U2OS cells transiently transfected with an empty luciferase vector (pGL3-basic), a CST6 promoter construct (-959/+30), or -959/+30 with two putative TBX2 binding site mutations (-857 and -172, respectively) and co-transfected with an empty expression vector (pDEST) or a TBX2 expression construct (TBX2). (B) Relative luciferase activity of pGL3-basic or a CST6 promoter construct (-959/+30) in U2OS cells, co-transfected in the presence of either pDEST or a TBX2 expression construct (TBX2) and each treated with Scrambled control (Scr) or EGR1 siRNA (EGR1si). (C) RqPCR analysis showing CST6 expression in MCF7 cells treated with EGR1si or Scr. (D) Endogenous ChIP assay showing recruitment of both TBX2 and EGR1 to a proximal region of the CST6 promoter (-153/+30) in MCF7 cells. A distal promoter region (-1150/-933) served as a negative internal control, a rabbit IgG was used to show specificity for the respective pull downs and a sample of 2.5% cell lysate prior to pulldowns was used as an input. (E) ChIP assay of MCF7 cells transiently transfected with EGR1 siRNA (EGR1si) or scrambled control siRNA (Scr) showing recruitment of TBX2 and EGR1 to the CST6 promoter. The same negative controls were used as outlined in (D).

Using transcription factor binding site prediction programs (such as TFSearch) we identified two putative TBX2 binding sites in the -959/+30 region. However, following SDM of both sites we observed no abrogation of TBX2 repression, indicating that they were not required (Figure 2A). We suspected that CST6 may be repressed by TBX2 through an EGR1-dependent mechanism similar to another characterized target NDRG1 (7). Accordingly, siRNA knockdown of EGR1 in MCF7 cells (Supplementary Figure S2.A) completely abolished the ability of TBX2 to repress the -959/+30 reporter (Figure 2B), accompanied by upregulation of CST6 mRNA (Figure 2C). However, we could not definitively show exactly which EGR1 site was responsible since SDM of the four most conserved EGR1 sites did not completely abolish TBX2-mediated repression (Supplementary Figure S2.B). We were, however, able to localize both TBX2 and EGR1 to the CST6 proximal promoter by ChIP assay (Figure 2D), with a region immediately upstream of the proximal promoter (-1150/-933) serving as an internal negative control. We could also show the requirement for EGR1 in this repression mechanism as siRNA knockdown of EGR1 (Supplementary Figure S2.C) resulted in a total loss of TBX2 recruitment to the CST6 promoter (Figure 2E). Together these data show that TBX2 represses CST6 through a proximal promoter region through an EGR1-dependent mechanism.

**Figure 3:**
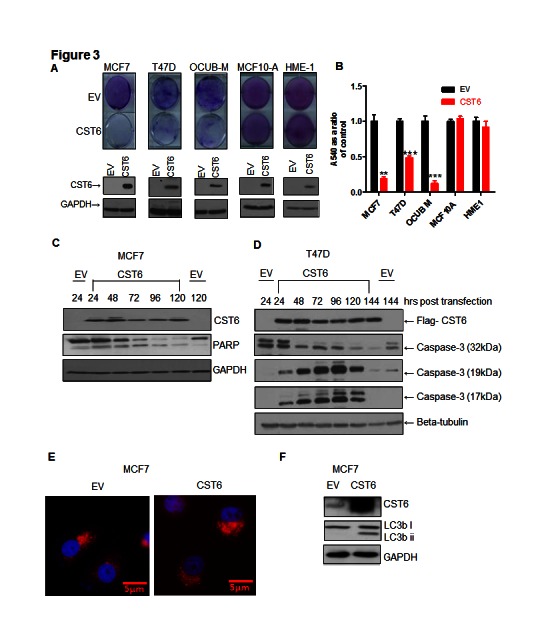
CST6 induces apoptosis in a cancer-specific manner (A) Crystal violet staining of MCF7, T47D, OCUB-M, MCF10A and HME1 cells transfected with either empty vector (EV) or the CST6 expression construct (CST6). The cells were grown for 6 days, stained with crystal violet to demonstrate cell viability. Matched Western blots showing CST6 expression compared to EV control with GAPDH antibody used as a loading control are also shown. (B) Crystal violet reabsorption of the plates stained in (A). (C) Western blot showing exogenous expression of CST6 in MCF7 cells collected over a 5 day time course alongside the corresponding EV and the consequent cleavage of PARP. GAPDH expression was used as a loading control. (D) Western blot analysis showing exogenous expression of CST6 in T47D cells collected over a 6 day time course alongside the corresponding EV and the consequent expression of full length caspase-3 (32kDa) and cleaved caspase-3 fragments (19kDa and 17kDa). Beta-tubulin expression was used as a loading control. (E) Immunofluorescence staining of MCF7 cells transiently transfected with either EV or CST6 expression construct and stained with 100nM Lysotracker. (F) Western blot analysis showing MCF7 cells transiently transfected with EV or CST6 expression construct (CST6), probed with a CST6, an LC3b i/ii antibody followed by a GAPDH antibody serving as a loading control.

We have previously shown that TBX2 expressing breast cancer cells were addicted to TBX2 and therefore acutely sensitive to TBX2 downregulation, resulting in dramatic growth inhibition and apoptosis [7]. We wanted to assess the contribution of CST6 re-expression to this cell-killing phenotype. Exogenous expression of CST6 resulted in dramatic cell death in TBX2-expressing cells with non-tumorigenic cells totally unaffected (Figure 3A, viability decreased by up to 90%, Figure 3B). The most dramatic cell kill effect was observed in the OCUB-M cell line. This cell line expresses mutant p53 and is ERα negative. In our initial screen of breast cancer cells we found the OCUB-M cells to be amongst those having an aberrant overexpression of TBX2. Even though these cells do not appear to have TBX2 levels comparable with MCF7 cells [7], they appear to be clearly addicted to the expression of TBX2 to a far greater extent, displaying a more dramatic cell killing phenotype following CST6 expression. We noted that CST6-induced cell growth inhibition was due to apoptosis since exogenous expression resulted in PARP cleavage of MCF7 cells (Figure 3C) and caspase-3 cleavage of T47D could be detected as early as 24 hours post transfection (Figure 3D). CST6 is known to be localized to lysosomes but we observed no loss of lysosomal integrity on specific lysosomal staining following CST6 overexpression in MCF7 cells (using LysoTracker Red DND-99 dye, Figure 3E, with a matched western blot shown in Supplementary Figure S3.A) Autophagy is known to act as a prelude to apoptosis following specific stimuli [21], characterized by the accumulation of microtubule-associated protein 1 light chain 3-phosphatidylethanolamine (LC3BII), a protein associated with the autophagosome membrane. The amount of LC3BII increased significantly upon CST6 overexpression in MCF7 cells (Figure 3F) indicative of the presence of autophagy. We therefore conclude that CST6 induces an autophagy related series of events culminating in the apoptosis of breast cancer cells.

**Figure 4:**
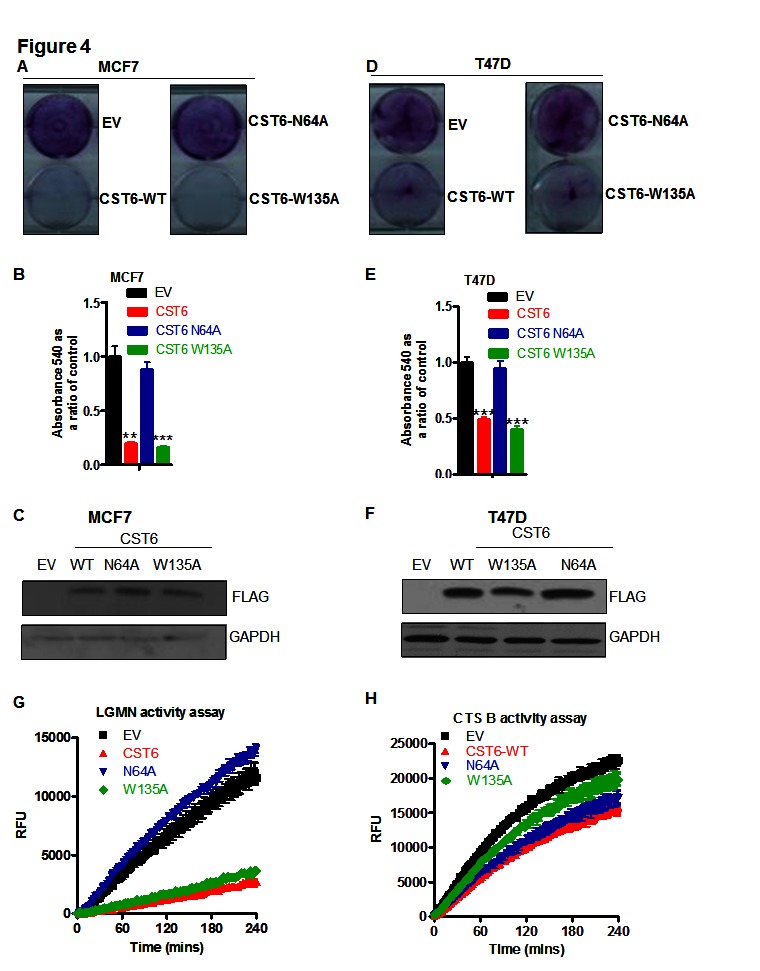
CST6 induces apoptosis through inhibition of asparaginyl endopeptidase activity (A) Crystal violet staining of MCF7 cells transfected with empty vector (EV) or WT-FLAG-tagged CST6 (CST6 WT) or FLAG-tagged mutants CST6-N64A and CST6-W135A. (B) Crystal violet reabsorption of the same samples as (A)(i) (C) Western blot of samples in (A) probed with a FLAG antibody and a GAPDH antibody serving as a loading control. (D) Crystal violet staining of T47D cells transfected with either EV or Wild type (WT)-FLAG-tagged CST6 or FLAG-tagged mutants CST6-N64A and CST6-W135A. (E) Crystal violet reabsorption of the same samples as (D). (F) Western blot analysis of the same samples as (D) probed with a FLAG antibody and a GAPDH antibody serving as a loading control. (G) LGMN activity assay showing relative fluorescence units (RFU) plotted as a function of time for the same samples as (A). (H) Cathepsin B activity assay showing RFU plotted as a function of time for the same samples as (A).

To compare cathepsin- versus AEP-inhibitory functions of CST6 for apoptosis induction we generated point mutants of CST6 previously shown to abrogate the respective inhibitory activities of CST6 [22]. Exogenous expression of FLAG-tagged wild-type CST6 (and a cathepsin inhibitory-defective point mutant (CST6-W135A) both resulted in significant apoptosis whilst the LGMN inhibitory-defective mutant (CST6-N64A) showed a complete loss of cell killing activity in both MCF-7 and T47D cells (Figures 4A and 4D, respectively) quantified as approximately 80% and 60% (Figures 4B and 4E). Western blotting confirmed that all three constructs showed equivalent expression (Figures 4C and 4F). LGMN activity assays in MCF-7 cells showed that while WT and CST6-W135A effectively inhibited LGMN activity, EV and the CST6-N64A mutant did not (Figure 4G). In contrast, the effect on Cathepsin B inhibition was weaker but the W135A mutant showed a clear reduction in Cathepsin B inhibition as expected (Figure 4H). Cathepsins (notably cathepsin B) have well described roles in proliferation and metastases. However, when we used the pan-cathepsin inhibitor, E-64-d, in the TBX2-expressing cell lines MCF7 and T47D we observed that complete inhibition of cathepsin activity did not result in any obvious growth difference or apoptosis (Supplementary Figure S4.A, quantified relative to control in S4.B), whilst a cathepsin B-specific assay showed that 10mM E-64-d resulted in complete inhibition of cathepsin B activity (Supplementary Figure S4.C). To specifically target cathepsin B an siRNA approach produced approximately a 90% knockdown (Supplementary Figure S4.D) but no corresponding cell growth inhibition (Supplementary Figures S4.E and S4.F) despite a corresponding 90% reduction in enzyme activity (Supplementary Figure S4.G). In fact cathepsin B knockdown actually resulted in the increased growth of MCF-7 cells, possibly reflecting the fact that it also plays a role in apoptosis via the Tumor Necrosis Factor α (TNFα) pathway.

**Figure 5:**
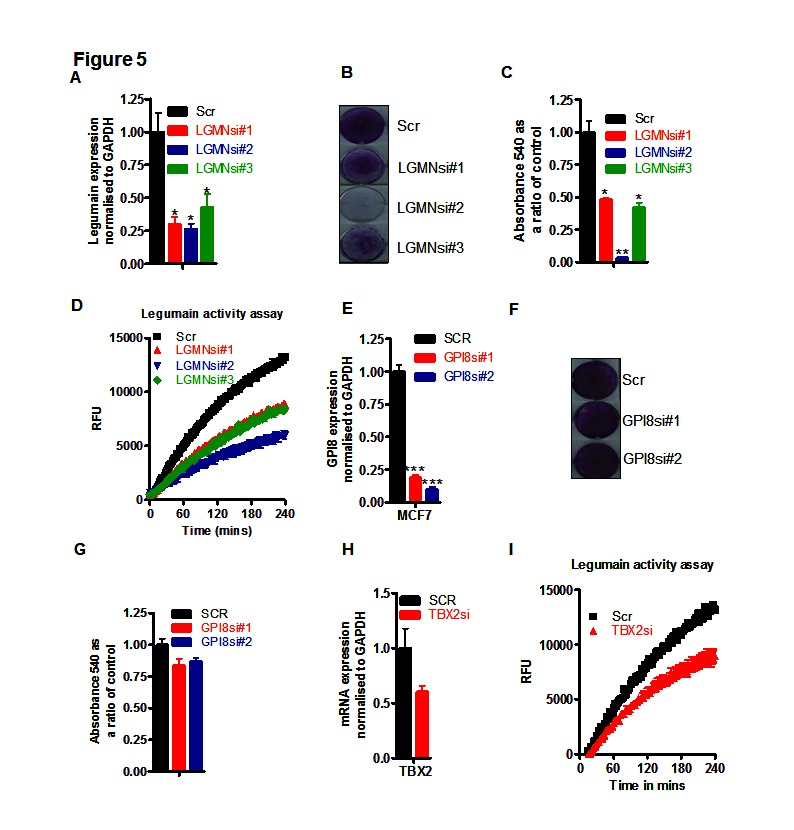
LGMN is the enzyme downstream of TBX2 and CST6 responsible for maintaining the proliferation of breast cancer cells (A) RqPCR of LGMN siRNA knockdowns (LGMNsi#1-3) in MCF7 cells compared with cells treated with a scrambled (Scr) control siRNA. (B) The same samples as (A) stained with crystal violet 5 days post transfection. (C) Crystal violet reabsorption of MCF7 cells from plates shown in (A). (D) LGMN activity expressed as RFU as a function of time in matched protein samples in MCF7 cells, treated with scrambled siRNA or the three different LGMN siRNAs #1 – #3 outlined in A. (E) RqPCR quantification of GPI8 siRNA knockdowns (GPI8si#1-&2) in MCF7 cells compared with cells treated with a scrambled (Scr) control siRNA. (F) Same samples as (E) stained with crystal violet 5 days post transfection. (G) Crystal violet reabsorption values of MCF7 cells shown in (E). (H) RqPCR showing TBX2 expression in MCF7 cells following knockdown with a scrambled siRNA (SCR) control or a TBX2 specific siRNA (TBX2si), normalized to endogenous levels of GAPDH. (I) LGMN activity assay matched to the experiment in (H).

The AEP (C-13) class of cysteine proteases contains only two proteins, namely, LGMN and glycosylphosphatidylinositol transamidase 8 (GPI8). Specifically targeting LGMN using three independent siRNA sequences, all produced significant reductions in LGMN mRNA (Figure 5A) coincident with a dramatic cell death phenotype (Figure 5B) of between 50-95% compared to scrambled control siRNA (Figure 5C). The efficacy of knockdowns reflected cell growth effects and knockdown of LGMN enzyme activities (Figure 5D). GPI8 knockdowns, however, (using two independent siRNAs, Figure 5E) failed to produce any significant apoptosis (Figure 5F, quantified <20% for both siRNAs in Figure 5G). Confirming that TBX2 expression levels indirectly controlled LGMN activity, we observed knockdown of TBX2 mRNA by siRNA (~40%) in MCF-7 cells (Figure 5H) was accompanied by a similar inhibition of LGMN activity (Figure 5I). Together these data suggest that the mechanism through which CST6 induces apoptosis is due to its inhibition of LGMN activity and that TBX2 drives tumor growth through maintenance of LGMN activity.

**Figure 6:**
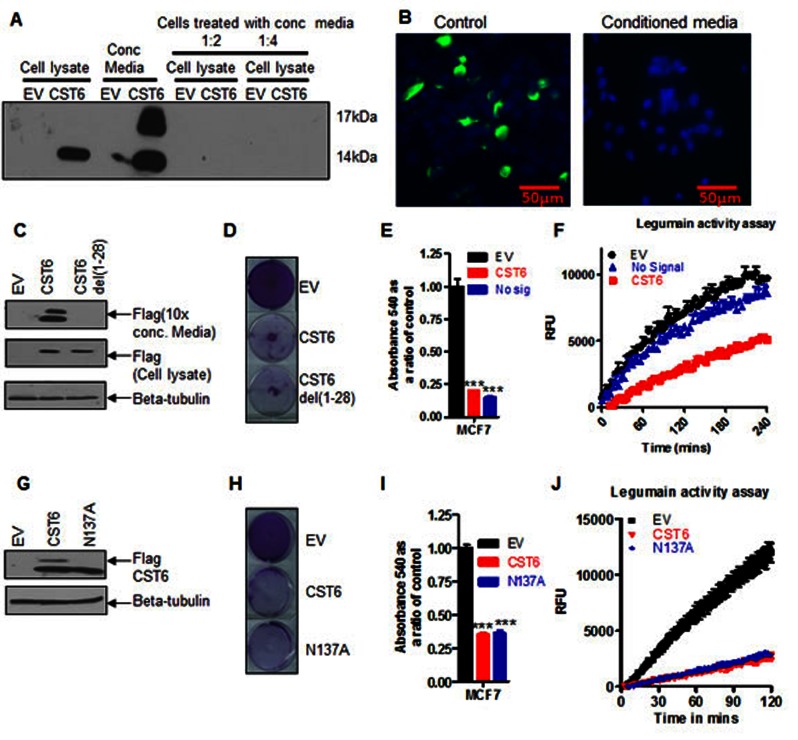
CST6 does not require secretion or glycosylation to inhibit LGMN activity (A) Western blot showing expression of FLAG-CST6 in MCF7 cells transfected with empty vector (EV) and or CST6 expression vector (lanes 1 and 2), concentrated media (lanes 3 and 4) and naive cells treated with a 1:2 dilution of EV and CST6 concentrated conditioned media (lanes 5 and 6), and a 1:4 dilution of EV and CST6 (lanes 7 and 8). (B) Immunofluorescence (10X) of MCF7 cells transfected with FLAG-tagged CST6 (left) and naive cells treated with 10X concentrated conditioned media for 24 hours post transfection and stained with FLAG antibody at 10X magnification (right). (C) Western blot showing conditioned media and cell lysates from MCF7 cells transfected with either EV or FLAG-CST6 or a FLAG tagged version of CST6del(1-28). Samples were collected 24 hours post transfection. Beta-tubulin was used as a loading control. (D) Crystal violet staining of viable cells 6 days post transfection of MCF7 cells with either EV or FLAG-CST6 or the mutant CST6 lacking the signal peptide CST6 del(1-28). (E) Crystal violet reabsorption of the samples outlined in (D) (F) LGMN activity assay of conditioned medium matched to the experiment (D) (G) Western blot showing glycosylated and unglycosylated forms of CST6 in MCF7 cells transfected with FLAG-CST6 or the glycosylation site mutant CST6 (N137A-CST6) 24 hours post transfection. Beta-tubulin was used as a loading control. (H) Crystal violet staining of MCF7 cells 6 days post transfection with either EV or WT-FLAG -CST6 or the glycosylation mutant form of CST6 (N137A). (I) Crystal violet reabsorption from the transfections outlined in (H). (J) LGMN activity assay of cell lysates matched to the experiment (H).

Many Cystatins are known to be secreted suggesting that the principal mode of CST6 action is extracellular. Following transfection of MCF7 cells with a FLAG-tagged CST6 we were able to detect two prominent forms of CST6 from cell media (Figure 6A). Whilst we could clearly visualize CST6 by immunofluroescence (IF) in directly transfected cells, we could not detect CST6 re-entry into naïve cells, even by incubating cells overnight with concentrated media (as shown by Western blotting, Figure 6A, or by IF, Figure 6B). There was also no evidence of cell killing observed following incubation with CST6-containing concentrated media (Supplementary Figures S5.A, S5.B and S5.C). We generated an N-terminal deletion mutant of CST6 lacking the signal peptide (CST6 del 1-28) which we could show was not secreted into media (Figure 6C) but was fully functional in cell killing (Figures 6D and E) indicating that CST6 secretion was not required for apoptosis. A LGMN activity assay of the lysate showed that CST6 del1-28 lost partial ability to inhibit CST6 but was still be to kill cells efficiently (Figure 6F). Several Cystatins (such as Cystatin F) are known to be glycosylated, an event which facilitates their entry into neighboring cells [23]. However, a FLAG-tagged CST6 mutant N137A was equally expressed alongside wild-type CST6 in transfected cells (Figure 6G), equally proficient in killing expressing cells (Figures 6H and 6I) and retained full inhibitory activity (Figure 6J). Together these data show that CST6 does not require secretion, cellular re-entry or glycosylation for the induction of apoptosis.

In order to see if our cell line data corresponded with tumor data we carried out immunohistochemical staining on Tissue Microarrays generated from a small cohort of breast tumors. In agreement with the cell line data we noted that CST6 expression inversely correlated with TBX2 expression (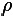-0.365,p-value =0.005). We observed that tumors expressing TBX2 showed an increased tendency towards metastasis while CST6 expressing tumors showed a trend towards a decrease in metastatic potential. In small subset of five TBX2-expressing tumors where chemoresponse data was available, we observed a modest trend towards chemoresistance. We also compared our data with the larger publically available datasets from *The Cancer Genome Atlas* (*TCGA*). In line with previous findings, CGH studies showed that TBX2 appeared to be amplified in approximately 10% of breast cancers. Microarray and RNAseq studies showed a consequent upregulation of TBX2 mRNA in these tumors (dataset from [24], and a provisional dataset currently in press, Nature). CST6 expression, if altered, was generally downregulated observed in about 5% of the tumors in both datasets. Tumors with altered TBX2 and CST6 expression also showed very poor overall survival by microarray studies (Supplementary figure S6.A).

## DISCUSSION

In this study we show for the first time that the putative breast tumor suppressor gene CST6 is consistently repressed by the oncogenic transcription factor TBX2 through a mechanism involving EGR1. Exogenous expression of wild-type CST6 in TBX2 expressing breast cancer cells resulted in significant apoptosis whilst non-tumorigenic breast cells remain unaffected. We show through a number of alternative approaches (siRNA knockdowns, pan-cathepsin inhibition and SDM) that CST6-induced apoptosis is due to its ability to specifically inhibit the AEP protease LGMN and not cathepsin-type proteases. Accordingly, LGMN activity assays confirmed that the observed CST6 growth effects were consistent with specific inhibition of LGMN. Whilst both LGMN and CST6 are known to be secreted proteins, neither the secretion nor glycosylation of CST6 was required for its cell killing function. We conclude that through its ability to counteract LGMN activity, CST6 represents an important barrier to breast tumorigenesis, a function which is abrogated by TBX2 transcriptional repression. These findings have obvious implications for breast tumorigenesis and highlight potential therapeutic opportunities for the treatment of TBX2 overexpressing or CST6-deificient breast cancers.

Aberrant TBX2 expression is likely to be just one of many pathways leading to loss of CST6 in cancers. CST6 loss in breast cancers has been shown to be associated with loss of both ERα and the progesterone receptor [25]. One of the mechanisms of CST6 downregulation is promoter methylation which was shown to be strongly associated with poor prognosis in operable breast cancers [26]. Indeed CST6 was amongst the 10 most hypermethylated genes in comparisons between breast cancers and matched normal breast tissues [27]. The mechanism responsible for CST6 promoter methylation is still unknown but it has been reported to be associated with aberrant AKT1 activation and disablement of the negative AKT regulator INPP4B in breast epithelial cells [28]. It has also been shown that hypermethylator breast cell lines which overexpress total DNA methyltransferase (DNMT) activity (and in particular DNMT3b) had significantly reduced levels of a number of genes including CST6 [29]. Whilst there is no evidence for TBX2 recruitment of DNMTs to target promoters, the C-terminal region of TBX2 has been shown to coimmunoprecipitate with HDAC1 in mouse melanoma cells to target repression of the key cell cycle inhibitor p21^WAF1^ [4]. TBX2 is also known to interact with histone H3 and to colocalize with regions of pericentric heterochromatin [30]. We hypothesize that TBX2 acts to target EGR1-responsive genes to facilitate histone methylation and deacetylation and to enforce a non-permissive heterochromatin microenvironment around promoters such as CST6.

LGMN expression is known to correlate with a lower rate of apoptosis, and increased metastasis and tumor invasion [31]. Whilst it correlates with poor prognosis in multiple tumor types [18, 19, 32, 33], the role of LGMN in promoting tumorigenesis remains a mystery. One downstream pathway which may be involved is the Matrix Metalloproteinase 2 (MMP2) pathway. LGMN is known to cleave pro-MMP2 leading to its activation, even in the presence of its natural inhibitor TIMP-2 [34]. LGMN has been shown to colocalize with integrins, at the cell surface ‘invadopodia’ of invading cells where it participates in the cleavage and activation of MMP2 to help drive invasion and metastasis [31]. We propose that the loss of CST6 expression observed in breast and many other solid tumor types would lead to elevated LGMN activities, elevated MMP2 signaling and enhanced proliferation, invasion and metastasis, reflecting the poor prognosis of LGMN expressing tumors.

Whilst the hallmarks of cancer are multifactorial, there are numerous examples demonstrating how aberrations in key oncogenes are clear driver events in cancer pathogenesis. Activation of other oncogenes may be rate-limiting events responsible for specific phases of cancer development such as initial transformation events whilst later events such as the progression or maintenance of tumor viability may require completely different oncogenic mechanisms [35]. Additionally, tumor cells may also be addicted to particular biological processes such as oncogenic Ras-mediated transformation and tumor growth being dependent on autophagy [36]. From a breast cancer perspective, a prime example of how to effectively target oncogene addiction is demonstrated by the success of trastuzumab treatment of HER2 positive breast cancers. The apparent ‘addiction’ to LGMN for cell survival that we have observed in breast cancer cell lines and the high activity of LGMN previously reported across a range of cancer types has made targeting this enzyme an attractive prospect for the development of novel inhibitors. Potent and selective LGMN inhibitors of *Schistosoma mansoni* and pig kidney LGMN have previously been developed [37]. Cell permeable, potent inhibitors have also been designed against recombinant mouse LGMN [38]. There have been a number of alternative therapeutic strategies developed to exploit the hyperactivity of LGMN in different tumor types. For example, elevated tumor LGMN activity has recently been used as the basis for developing LGMN-cleavage strategies to activate Auristatin prodrugs [39], in addition to more conventional chemotherapeutic agents such as etoposide [40] or doxorubicin [31]. The doxorubicin prodrug completely arrested the growth of a variety of neoplasms, including multidrug-resistant tumors *in vivo* and significantly extended survival without evidence of myelosuppression or cardiac toxicity [41]. In addition liposomal nanoparticles encapsulating doxorubicin coupled to a LGMN inhibitor showed high potency, good stability and little cross reactivity with other cysteine proteases [42]. The prevalence of CST6 loss and LGMN hyperactivation across many tumor types therefore represents an exciting opportunity for the development of novel targeted therapies.

In summary, we have identified a novel pathway regulated by the breast cancer oncogene TBX2. We show for the first time that TBX2 mediated transcriptional repression of CST6 is an important event for maintaining the tumorigenesis of a subset of breast cancers, through aberrant activation of the protease LGMN. These findings provide an opportunity for the development of specific targeted treatment for multiple tumor types, linking an imbalance of Oncogene-Tumor Suppressor expression (TBX2-CST6) with a defined enzymatic hyperactivity (LGMN). We suggest that the development of cell permeable LGMN inhibitors may be an important advance in the clinical management of poor prognosis breast cancers and multiple other tumor types.

## MATERIALS AND METHODS

### Maintenance of cell lines:

Full details of the maintenance of the MCF7, T47D, BT474, U2OS and MCF7-DN cells are provided in [7]. OCUB-M cells were maintained in DMEM media supplemented with 10% fetal calf serum, 1mM sodium pyruvate (Life Technologies, Inc, Paisley, UK). MCF10A cells were cultured in DMEM:F-12 1:1 phenol red free medium, 5% Horse Serum, 20ng/ml EGF, 500ng/mlHydrocortisone, 100ng/ml Cholera toxin, 10ng/ml insulin and 1mM L-glutamine. HME-1 cells were cultured in Medium 171 supplemented with an MGEM bullet kit (Cascade Biologicals, Life Technologies) and 5 micrograms/ml transferrin (Sigma-Aldrich, Dorset, UK). All were grown in 5% CO_2_ in a humidified incubator. For E-64d treatment of cells, a stock solution was prepared at 5 mg/ml, MeOH:H2O (1:1) and cells were treated at a final concentration of 10μM.

### Luciferase assay:

Luciferase assay was carried out in U2OS cells transfected with control (pGL3-basic empty vector) or the vector containing the CST6 promoter constructs. The assay was carried out as previously described [7].

### Chromatin immunoprecipitation:

This assay was carried out in MCF7 cells. The method is described in [7]. Promoter primers were designed against a proximal region and a region approximately 1000bp upstream of the translational start site as a non-specific negative control (primer sequences are detailed below).

### Enzyme activity assays:

MCF7 and T47D cells were harvested in PBS at 4ºC. Cell pellets were re-suspended in lysis buffer (40mM citric acid, 121mM NaH2PO4, 1mM EDTA, 0.1% CHAPS, 1mM DTT, pH5.8) on ice for 15 mins, syringed through a 21-gauge needle and frozen at -80ºC. Cells were then centrifuged at 16000g for 20 mins at 4ºC and the protein concentration in the supernate was determined. Activity of cellular enzyme was measured by diluting lysate in assay buffer (40mM citric acid, 121mM NaH2PO4, 1mM EDTA, 1mM DTT, pH5.8). The reaction for LGMN was initiated by addition of 10 microlitres of the substrate Z-Ala-Ala-Asn-MCA (Peptides Institute, Osaka, Japan) to a final concentration of 10 micromolar substrate. The reaction for Cathepsin B was initiated by the addition of 10 microlitres of the substrate Ac-RR-AFC (Bio Vision, San Francisco, CA, USA).The plates were read on the Synergy 4 microplate reader (Biotek, Bedfordshire, UK) at 37 ºC and the readings were recorded at excitation 360nm and emission 460nm every two minutes. Data was blanked against a substrate only well in Gen 5 software. For the cell free assay, rLGMN (R&D systems, Abingdon, UK) was activated in the presence of 50mM NaOAc,100mM NaCl, pH 4.0 for 2 hours at 37ºC. 500nmol of activated rLGMN was diluted in 10mls of assay buffer.

Preparation of cells, reagents, western blotting procedures are described in [43], Real Time quantitative PCR (RqPCR), siRNA treatments and Chromatin Immunoprecipitation (ChIP) assays in [7] and cell proliferation assays in [44]. Sequences of RqPCR, luciferase, and ChIP primers as well as antibody sources and dilutions are listed in supplementary information. The details for construction of the tissue microarray blocks were described previously [45].

### Conflict of interest

The authors declare no conflict of interest.

## SUPPLEMENTARY FIGURES AND TABLES


