# Supplementary material for: TBX2 represses CST6 resulting in uncontrolled legumain activity to sustain breast cancer proliferation: a novel cancer-selective target pathway with therapeutic opportunities
Source: Oncotarget. 2014 Feb 8;5(6):1609–20. doi: 10.18632/oncotarget.1707 (PMC4057604; doi:10.18632/oncotarget.1707)
Supplement: Supplementary file 1 [file oncotarget-05-1609-s001.pdf]

SUPPLEMENTARY INFORMATION

PRIMER SEQUENCES

RQPCR PRIMERS

TBX2

F – 5’-GGCTTCAACATCCTAAACTCC-3’

R – 5’-AAGATCGACCAACAACCCGTTT-3’

CST6

F - 5’-GCAACAGCATCTACTACTTC-3’

R - 5’- AGTCTGTGCTCCCCATCTCC-3’

LGMN

F - 5’-GAACACCAATGATCTGGAGGA -3’

R **-** 5’- GGAGACGATCTTACGCACTGA -3’

GPI8

F - 5’-CGTCTTCTTTCTGATGACAG-3’

R - 5’- GCATCCGCGAGTTCTATG -3’

EGR1

F – 5’-TTTGCCAGGAGCGATGAA-3’

R – 5’-CCGAAGAGGCCACAACACTT-3’

GAPDH

F – 5’-GGGAAGGTGAAGGTCGGAGT-3’

R – 5’-CTGGTTTTAACTCTGGTAAAGTGGA- 3’

β-ACTIN

F – 5’- AGACCTGTACGCCAACACAG-3’

R – 5’-GGAGCAATGATCTTGATCTTCA-3’

<u>siRNA SEQUENCES</u>

Scrambled control siRNA- SCR-GFP

5’- AAGCAGCACGACUUCUUCAAG-3’

TBX2

5’- GGAGCUGUGGGACCAGUUC-3’

EGR1

5’- CCAUGGACAACUACCCUAA -3’

CST6

5’- CAGAACUCCUCUCAGCUCCUA -3’

LGMN#1

5’ - CCAUGGAUCUACUGGAAUA-3’

LGMN#2

5’ - GGACUACACUGGAGAGGAU - 3’

LGMN#3

5′- GGGCAUAGGAUCCGGCAAA - 3′

GPI8#1

5’- GUGGAAAUUACAACAGAGA -3’

GPI8#2

5’- GTGUUUAGUCACAAGAAUA -3’

CLONING PRIMERS

CST6

F- 5’- GCCATGGCGCGTTCGAACCTCCCG - 3’

R- 5’-CTACTTGTCATCGTCGTCCTTGTAGTCCATCTGCACACAGTTGTG- 3’

LUCIFERASE PRIMERS

CST6 PROMOTER (-959 to +30)

F - 5’ - CACTGCCTCGAGGCTAGAGCGCAGTGGTGCC-3’

R - 5’ – CACTGCAAGCTTGCCATGGCCGTCAGTGCCG-3’

CST6 PROMOTER (-189 to +30)

F- 5’ – CACTGCCTCGAGTCCCTCCCCGGGCCAGGTG-3’

R - 5’ – CACTGCAAGCTTGCCATGGCCGTCAGTGCCG-3’

SITE DIRECTED MUTAGENESIS PRIMERS

CST6 N64A

F - 5’-GCTACAACATGGGCAGCGCCAGCATCTACTACTTCC-3’

R - 5’-GGAAGTAGTAGATGCTGGCGCTGCCCATGTTGTAGC-3’

CST6 W135A

F- 5’-GGTCCTTGTGGTTCCCGCGCAGAACTCCTCTCAGC-3’

R- 5’-GCTGAGAGGAGTTCTGCGCGGGAACCACAAGGACC-3’

CST6 N137A

F -5’-CTTGTGGTTCCCTGGCAGGCCTCCTCTCAGCTCCTAAAG-3’

R - 5’-CTTTAGGAGCTGAGAGGAGGCCTGCCAGGGAACCACAAG-3’

CST6 PROMOTER TBX2#1

F - 5’-CTGAGTAGCTGGGAATTCAGGCTCGTGCCACCACGCCTGGCT-3’

R - 5’-AGCCAGGCGTGGTGGCACGAGCCTGAATTCCCAGCTACTCAG-3’

CST6 PROMOTER TBX2#2

F - 5’-CCTCTCCCTCCCCGGGCCAGGCTCGTCCTGGAGGGCAGGGAA-3’

R - 5’-TTCCCTGCCCTCCAGGACGAGCCTGGCCCGGGGAGGGAGAGG-3’

CST6 PROMOTER EGR1#1

F - 5’-CGTCTAGCTCTGTCGTTTAGGCTGGAATGCTGC-3’

R - 5’-GCAGCATTCCAGCCTAAACGACAGAGCTAGACG-3’

CST6 PROMOTER EGR1#2

F- 5’-ATTACAAGCATGCCTTTTTGCCTGGCCAGGTAG-3’

R - 5’-CTACCTGGCCAGGCAAAAAGGCATGCTTGTAAT-3’

CST6 PROMOTER EGR1#3

F- 5'- GTGGCAACAGTAAGATTTCACGAAGAGCTGCGG-3'
R- 5'- CCGCAGCTCTTCGTGAAATCTTACTGTTGCCAC-3'

CST6 PROMOTER EGR1#4
F - 5'CCGCCCCAGCTCCAGGCCGCGTTTGCGCATCGCGGGCGTCGGGCG-3'
R - 5'CGCCCGACGCCCGCGATGCGCAAACGCGGCCTGGAGCTGGGGCGG-3'

ChIP PCR PRIMERS
CST6 CHIP- (-153 to +30)
F - 5'-AAG CGT CTT GGC ACG CGG GTG CGC-3'
R – 5'- CACTGCAAGCTTCAGAGCCGTGAGTGCCGAGC-3'

CST6 -1150 (-1150 to -933)
F - 5'-CAG TTT GTG TCC TGT GGG CTC AGT-3'
R - 5'-CAC CAC TGC GCT CTA GCC TG-3'

Supplementary Table 1
Table listing the different antibodies used throughout the paper. The company where they were procured from and the details of the dilution they were used at.

Antibody	Company	Catalogue Number	Species	Dilution	Molecular weight
**FLAG M2**	Sigma	F184	Mouse	1:3000	N/A
**TBX2**	Upstate	07-318	Rabbit	1:1000	74kDa
**EGR1**	Cell Signalling	#4154	Rabbit	1:1000	75kDa
**CASPASE-3 (Cleaved)**	Cell Signalling	9661L	Rabbit	1:1000	19kDa<br>17kDa
**CASPASE 3**	Merck	AM39	Mouse	1:1000	32kDa
**PARP**	eBiosciences	14-6666	Mouse	1:5000	116kDa<br>85kDa
**β-TUBULIN**	Sigma	T4026	Mouse	1:3000	55kDa
**GAPDH**	Biogenesis	4699-9555	Mouse	1:5000	36kDa

Supplementary figures:

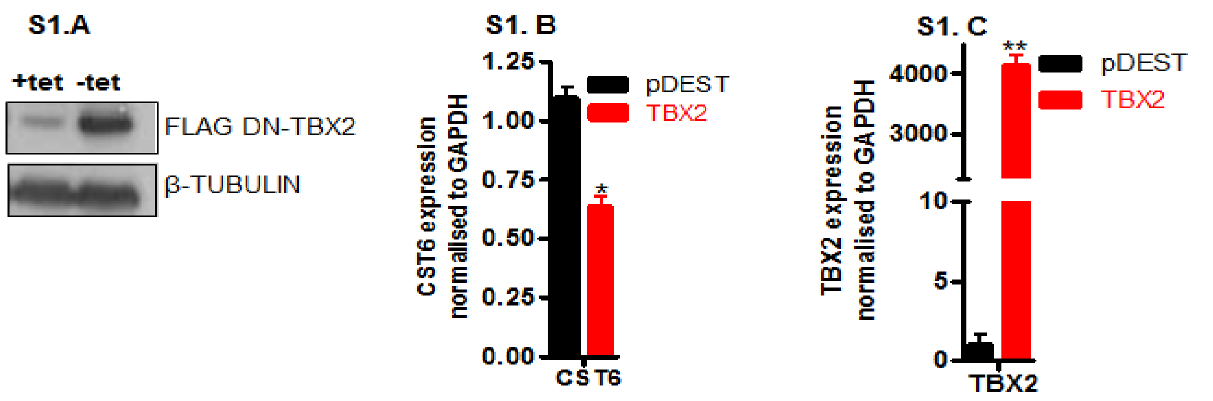

(S1.A) Western blot showing MCF7 cells stably transfected with a tetracycline responsive FLAG tagged Dominant Negative TBX2 (Flag DN-TBX2). β-tubulin served as a loading control. **(S1.B)** Bar graph of real time PCR analysis showing CST6 mRNA levels in U2OS cells transiently transfected with empty vector (EV) or vector containingTBX2. **(S1.C)** Real time PCR showing TBX2 overexpression in the same samples as (S1.A). GAPDH mRNA was used for normalization.

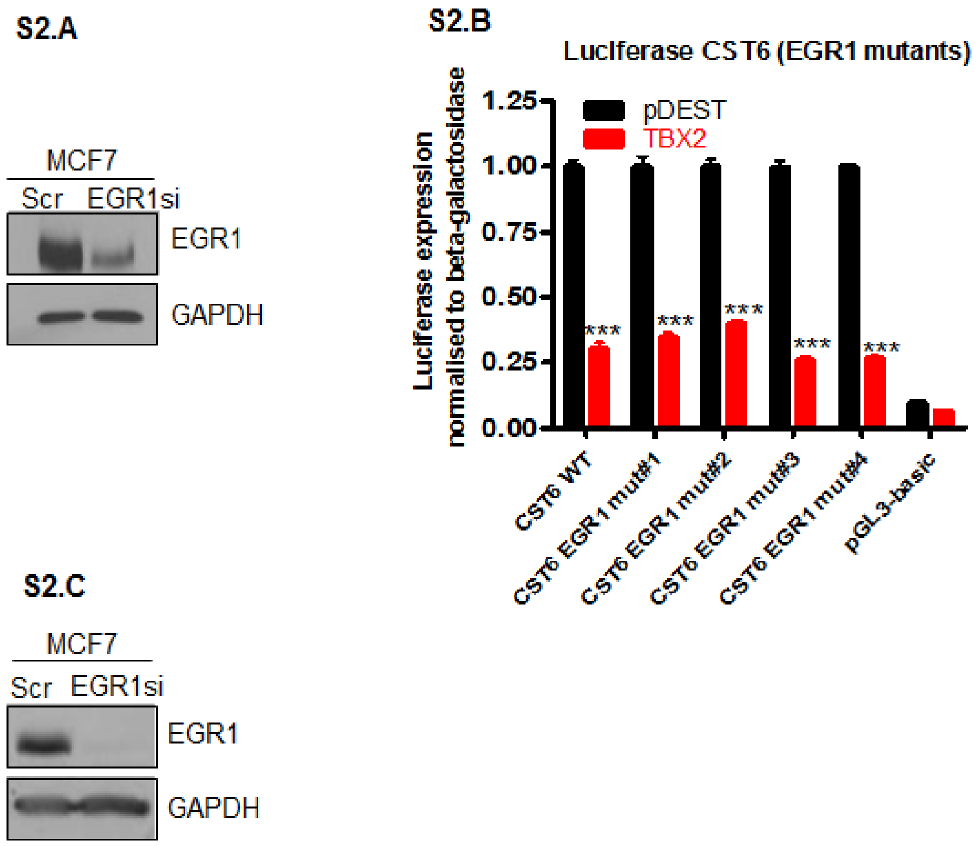

(S2.A) Western blot of U2OS cells treated with Scrambled control (Scr) or EGR1 siRNA (EGR1si) with GAPDH antibody used as a loading control. **(S2.B)** Bar graph showing luciferase reporter assay of U2OS cells transiently transfected with an empty luciferase vector (pGL3-basic) or the same vector containing the CST6 promoter construct (-959 to +30), or the same construct with mutations in the four putative EGR1 binding sites. All four vectors were co-transfected with either an empty expression vector (pDEST) or the same vector containing TBX2 expression constructs (TBX2). **(S2.C)** Western blot of MCF7 cells transiently transfected with EGR1 siRNA (EGR1si) or scrambled control siRNA (Scr) with GAPDH antibody used as a loading control.

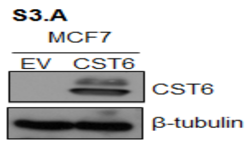

(S3.A) Western blot of MCF7 cells transiently transfected with either EV or CST6 expression construct. GAPDH expression was used as a loading control

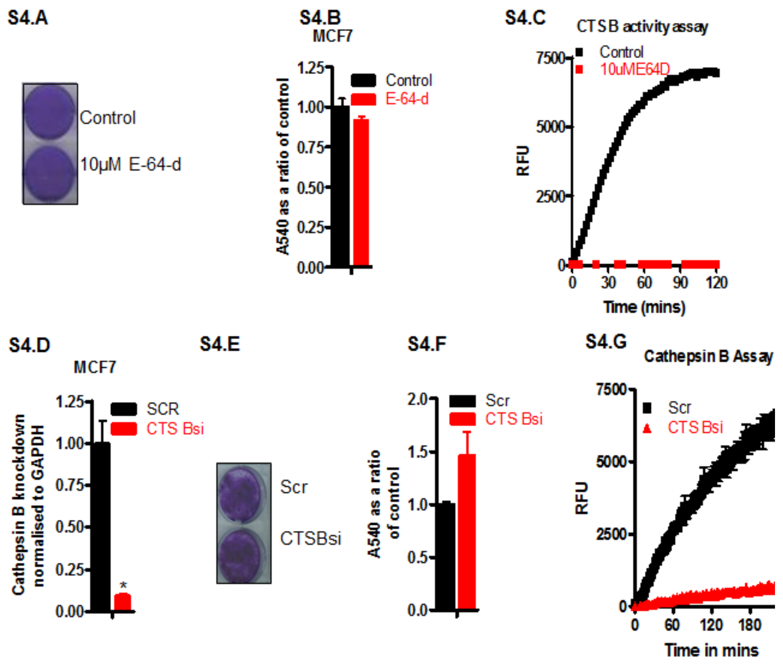

(S4.A) Crystal violet staining of MCF7 cells treated with the pan-Cathepsin inhibitor E-64-d. **(S4.B)** Crystal violet reabsorption of the samples outlined in (S4.A). **(S4.C)** Cathepsin B activity assay showing RFU plotted as a function of time for the same samples as (S4.A).

(S4.D) Bar graph of real time PCR analysis showing CTS-B mRNA levels in MCF7 cells following treatment with CTS-B siRNA or a scrambled control. **(S4.E)** Scanned images showing Crystal violet staining of MCF7 cells treated CTS-B siRNA or a scrambled control. **(S4.F)** Bar graph of crystal violet reabsorption of the samples outlined in (S4.E). **(S4.G)** Cathepsin B activity assay showing relative fluorescence units (RFU) plotted as a function of time for the same samples as (S4.E).

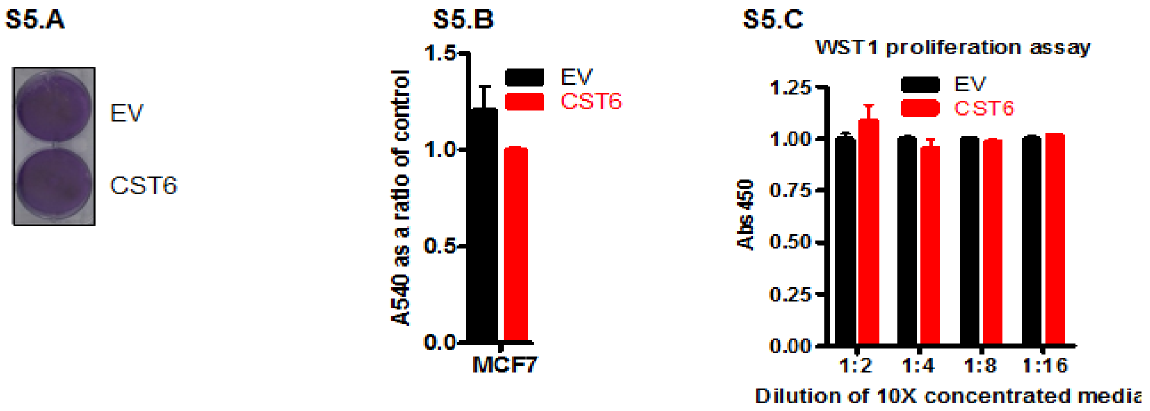

(S5.A) Crystal violet staining showing number of viable cells 6 days after treatment with undiluted 10X concentrated EV and CST6 conditioned media supplemented with 10% serum. **(S5.B)** Crystal violet reabsorption values of naïve MCF7 cells treated with dilutions of conditioned media from (S5.A). **(S5.C)** WST-1 cell proliferation assay performed on cells 6 days post treatment with 10X concentrated conditioned media taken from cells transfected with EV and CST6. Conditioned medium was diluted with fresh medium in the rations 1:2, 1:4, 1:8 and 1:16.

S6.A

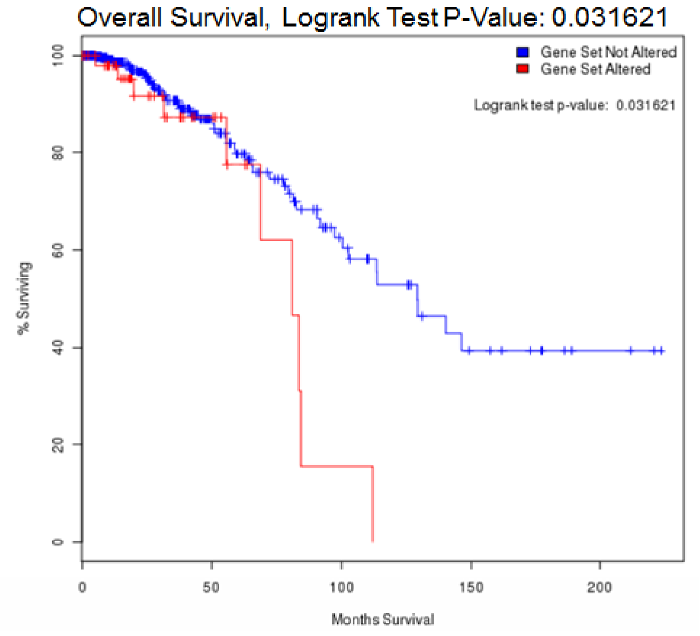

(S6.A) Overall survival curve comparing altered TBX2-CST6 mRNA expression levels (microarray z-scores), in 825 cases with the unaltered dataset from the breast invasive carcinoma data (TCGA, Nature 2012).
